# Frequent PD-L1 expression in oral squamous cell carcinoma of non-smokers and non-drinkers, and association of tumor infiltrating lymphocytes with favorable prognosis.

**DOI:** 10.1016/j.tranon.2025.102357

**Published:** 2025-03-15

**Authors:** FJ Mulder, EJ de Ruiter, TFB Gielgens, F Farshadpour, R de Bree, MFCM van den Hout, B Kremer, SM Willems, EJM Speel

**Affiliations:** a Department of Otorhinolaryngology and Head & Neck Surgery, GROW-School for Oncology and Developmental Biology, Maastricht University Medical Center, Maastricht, Netherlands; b Department of Pathology, University Medical Center Utrecht, Utrecht, Netherlands; c Department of Otorhinolaryngology, Amsterdam University Medical Center, Amsterdam, Netherlands; d Department of Otorhinolaryngology, BovenIJ Hospital, Amsterdam, Netherlands; e Department of Head and Neck Surgical Oncology, University Medical Center Utrecht, Utrecht, Netherlands; f Department of Pathology, GROW-School for Oncology and Developmental Biology, Maastricht University Medical Center, Maastricht, Netherlands; g Department of Otorhinolaryngology and Head & Neck Surgery, Erasmus Medical Center, Rotterdam, Netherlands; h Department of Pathology and Medical Biology, University Medical Center Groningen, Groningen, Netherlands; i Department of Pathology and Clinical Bioinformatics, Erasmus Medical Center, Rotterdam, Netherlands

**Keywords:** Head and neck cancer, Programmed death ligand 1, Tumor infiltrating lymphocytes, Non-smokers, Non-drinkers

## Abstract

**Objectives:**

To determine the presence and prognostic value of PD-L1 and PD-L2 expression, and tumor-infiltrating lymphocytes (TILs) in oral squamous cell carcinoma (OSCC) of non-smokers and non-drinkers (NSND).

**Materials and methods:**

Clinical characteristics and tumor tissue of 86 NSND with OSCC were retrospectively collected and analyzed for protein expression on tissue microarrays. Immunohistochemistry was performed for expression of PD-L1 CPS, PD-L2 TPS, and PD-1, CD45, CD8, CD4, CD3, and FoxP3-positive TILs/mm^2^. Slides were digitally evaluated using QuPath. Differences in 5-year DFS and OS were determined by log rank analysis. Predictors for survival were determined by multivariable cox regression analysis.

**Results:**

Eighty-eight percent (76/86) of OSCC showed PD-L1 expression (CPS ≥1). Patients with high numbers of CD4-positive TILs showed a better DFS and OS than patients with low numbers of CD4-positive TILs. In the best multivariable model, CD4-positive TILs were an independent predictor for DFS (*p* = 0.010) and OS (*p* = 0.002) too. Additionally, patients with high numbers of CD45-positive TILs and a high CD8/FoxP3 ratio showed a better OS, of which the CD8/FoxP3 ratio was a near significant independent predictor (*p* = 0.050). Over 40 % of OSCC were PD-L1+/TIL+.

**Conclusion:**

A large number of OSCC in NSND show PD-L1 expression (CPS ≥1). CD4 was a significant predictor for DFS and OS, in addition to the CD8/FoxP3 ratio being a near significant predictor for OS. The combination of frequent high CD8-positive TIL infiltrates in PD-L1-positive tumors makes NSND with OSCC in theory interesting candidates for treatment with immune checkpoint inhibitors.

## Highlights

• >85 % of OSCC in non-smokers and non-drinkers show PD-L1 expression (CPS ≥1).
• There are frequent high CD8-positive TIL infiltrates in PD-L1-positive OSCC.
• CD4-positive TILs predict disease free survival and overall survival.

## Introduction

Although most head and neck squamous cell carcinomas (HNSCC) result from tobacco smoking or excessive alcohol consumption, there are patients without exposure to these traditional risk factors. In these non-smokers and non-drinkers (NSND), the oncogenic viruses human papillomavirus (HPV) and Epstein-Barr virus (EBV) appear to play a significant role in oropharyngeal squamous cell carcinoma (OPSCC) and nasopharyngeal squamous cell carcinoma, respectively [1] However, HNSCC in NSND typically develops in the oral cavity, where HPV-prevalence is both very low and does not affect the outcome as it does in HPV-positive OPSCC [[2], [3], [4], [5], [6]] Instead, it has been suggested that there are differences in the microenvironment of these oral squamous cell carcinoma (OSCC) [[7], [8], [9]] In 2019, the Food and Drug Administration (FDA) approved the use of treatments affecting the interplay between the tumor and its microenvironment in the form of anti-PD-1 immune checkpoint inhibitors (ICIs) in first and second line palliative care for recurrent or metastatic HNSCC [10] Also, there is evidence that neoadjuvant treatment with ICIs could have a major pathological response at the primary tumor site [11] In this context, since the standard of care in HNSCC of NSND frequently involves surgery because of the predominant tumor localization in the oral cavity, analysis of immune cells affected by ICIs is interesting in this patient group.

Immune evasion by the tumor could emerge via activation of immune checkpoint cascades, when the programmed death-1 (PD-1) receptor on T-cells binds with either of its ligands on tumor cells: programmed death-ligand 1 (PD-L1) or programmed death-ligand 2 (PD-L2) [9,12] This can inhibit the activity of tumor-infiltrating lymphocytes (TILs), of which there are different subsets with different functions in the immune microenvironment. CD45 is a pan-leukocyte maker and CD3 a general T-cell marker. Cytotoxic T-lymphocytes (characterized by CD8 expression) can directly target and destroy tumor cells, which can be stimulated by T-helper cells (characterized by CD4 expression) [13] Regulatory T-cells (Tregs, characterized by FoxP3) on the other hand, can suppress an anti-tumor response by maintaining immunological tolerance to host tissues [13] When Tregs are scarce they are less likely to overshadow the function of cytotoxic T-cells, indicated by a high CD8/FoxP3 cell ratio [14] Based on PD-L1 expression and presence or absence of TILs, different tumor microenvironments have been proposed, of which patients with an “adaptive immune resistance” (PD-L1+/TIL+) microenvironment are thought to be the group that largely responds to ICIs [15]

The goal of this study was to determine the PD-L1 and PD-L2 expression, the presence of PD-1, CD45, CD8, CD4, CD3, and FoxP3-positive TILs, and the CD8/FoxP3 ratio in OSCC of NSND. Secondary analyses evaluated if the presence of these markers was a predictor of disease free survival (DFS) and overall survival (OS).

## Materials and methods

### Subjects

Consecutive patients with OSCC were selected in two university hospitals in The Netherlands. In the University Medical Center Utrecht, patients were retrospectively selected between 1980 and 2004, as described previously [2] In the Maastricht University Medical Center, OSCC patients have been selected retrospectively between 2011 and 2016. Inclusion criteria were: ≥18-years-old NSND patients with a primary OSCC, available formalin-fixed, paraffin-embedded (FFPE) tumor tissue, and >3 years follow up. Patient characteristics, risk factors, World Health Organization tumor classification, AJCC seventh edition staging, and information concerning recurrent disease or death were collected from the medical records. Non-smoking was defined as having no history of smoking, non-drinking as having no history of alcohol consumption (not even ‘sporadic’ alcohol consumption), as reported in the patients’ medical records during both their first presentation at the Head and Neck outpatient clinic, and during the pre-anesthesia screening before panendoscopy or surgical resection. Patients with a second primary tumor in the head and neck region, tumors outside the upper aerodigestive tract, a cervical metastasis of unknown origin, or a histopathologic diagnosis other than squamous cell carcinoma were excluded.

The Medical Ethics Review Committee of the MUMC (2017–0300) has approved this study and the principles outlined in the Declaration of Helsinki were followed. All data and tissues were handled according to General Data Protection Regulation.

### Tissue microarrays and immunohistochemistry

FFPE blocks were retrieved from the departments of Pathology (MPTC PA1711-path-114) and hematoxylin and eosin sections were digitally evaluated with a senior head and neck pathologist (SW or MH), using Pannoramic viewer (3DHISTEC, Budapest, Hungary). Per patient, three 0.6 mm tumor tissue cores and one normal epithelium core were selected and placed in three tissue microarrays (TMAs).

**Fig. 1:**
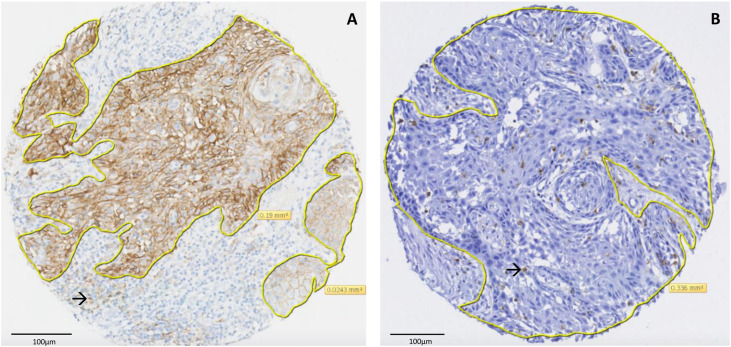
Example of a tissue micro array core with staining for PD-L1 (**A**, combined positive score (CPS) = 100) and CD45 (**B**, 150 tumor infiltrating lymphocytes (TILs)/mm^2^). The tumor has been delineated to separate it from the stroma and to determine the tumor area in mm^2^. For the PD-L1 CPS, the number of PD-L1 positive cells in the core (viable invasive tumor cells, lymphocytes (example marked with →), macrophages) were divided by the total number of viable invasive tumor cells, and multiplied by 100. For CD45, the number of positive lymphocytes (example marked with →) within the delineated area were divided by the total tumor surface area. Images were digitally evaluated at 200x magnification as shown, an area of 100µm is marked in both images.

Five µm FFPE TMA sections were subjected to immunohistochemistry (IHC), using primary antibodies directed against PD-L1, PD-L2, PD-1, CD45, CD8, CD4, CD3, and FoxP3 (Supplementary Table 1). For PD-L1, the standardized SP263 assay was performed on the Ventana Benchmark Ultra platform (Ventana Medical Systems, Tucson, AZ). The other immunostainings were performed on a Dako Omnis autostainer (Agilent Technologies, Carpinteria, CA) using the EnVision FLEX+ Mouse (LINKER) kit. In short, antigen retrieval was performed with a sodium-citrate solution (pH 6.0) or a high-pH buffer (pH 9.0). Endogenous peroxidase was blocked with Dako REAL Peroxidase-Blocking Solution prior to 20 min of incubation with the primary antibody. Binding of the antibodies was visualized by an enzymatic reaction with horseradish peroxidase and 3,3′-Diaminobenzidine as substrate, producing a brown precipitate (Fig. 1, Supplementary figure 1). Slides were counterstained with hematoxylin, scanned with a Pannoramic 1000 digital slide scanner (3DHISTEC, Budapest, Hungary) at 400x magnification, and digitally evaluated at 200x magnification using the open-source software QuPath v0.4 [16] Patients were excluded when <10 % of the surface area of the three tumor cores was available. Placental tissue was used as a positive control for PD-L1 and tonsil tissue for the other IHC markers.

### Evaluation of immunohistochemical staining

PD-L1 expression was evaluated using the combined positive score (CPS), which is the number of viable invasive tumor cells, lymphocytes, and macrophages with any cell membrane staining, divided by the total number of viable invasive tumor cells, multiplied by 100 [17] The final CPS per patient was the average score of its three tumor cores in the TMA, and ranged from 0 to 100. Since the FDA approval for the ICI pembrolizumab in HNSCC includes a CPS ≥1 as selection criterion, this cutoff was considered to be PD-L1-positive [10,17] Additionally, the cutoff ≥20 was determined [17] For PD-L2, the tumor area per TMA core was digitally delineated to calculate the tumor surface area. Next, the tumor proportion score (TPS) was determined by assessing the percentage of PD-L2-positive tumors cells (any cell membrane staining), taking the weighted average of the three TMA cores per patient depending on the tumor surface area. Scores ranged from 0 % to 100 %, and a ≥ 1 % cutoff was used to define a PD-L2-positive expression [12,18,19]

The number of PD-1, CD45, CD8, CD4, CD3, and FoxP3-positive TILs were divided by the digitally delineated tumor surface area (Fig. 1). Here, the weighted average of the three TMA cores was calculated as well to acquire a number of TILs/mm^2^ per patient. Van Kempen et al. defined the optimized cutoff value for CD8, CD4, and CD3 as a predictor of OS as either 100 or 150 TILs/mm^2^ [20] This was close to the optimal CD8 cutoff values of 138 TILs/mm^2^ and 182 TILs/mm^2^ in two other studies [21,22] Therefore, derived from the median number of TILs/mm^2^ per marker in this study, a cutoff of 100 TILs/mm^2^ was used for PD-1 and FoxP3, and 150 TILs/mm^2^ for CD45, CD8, CD4, and CD3. The CD8/FoxP3 ratio was determined using the TILs/mm^2^ and a high ratio was defined as ≥2.5 [23,24]

### Statistical analysis

Differences in clinical parameters between PD-L1, PD-L2, and TILs-positive and negative OSCC were evaluated by Mann-Whitney U test for age because of a non-normal distribution, Fisher's exact test for binominal variables, and the Fisher-Freeman-Halton exact test for categorical variables. The 5-year DFS and OS were estimated with Kaplan-Meier curves and differences were determined by log rank test. DFS was defined as the last date of treatment until the biopsy date of a histologically proven recurrence or second primary tumor in the head and neck region. OS was defined as the time between the primary tumor biopsy date and death. Censoring took place when patients were lost to follow-up, deceased without recurrent disease for DFS, or at the cutoff point of 60 months. To determine if TILs were independent predictors for survival, variables with a statistically significant (*p* < 0.05) or near significant (*p* < 0.10) relationship were evaluated in multivariable cox regression analysis using the backward conditional method for the patients who were treated with curative intend. Clinical variables that predicted survival were added to the model as well (T-stage, N-stage, and received therapy). Analyses were performed using IBM SPSS Statistics 25.0 (IMB corp., Armonk, NY).

Two independent assessors who were blinded for patients’ clinical characteristics scored all tumors for PD-L1 CPS and PD-L2 TPS. For the TILs, 1/7th of all TMA cores were randomly selected and evaluated by a second assessor. Inter-observer reliability was determined with the intraclass correlation coefficient (ICC), based on a single rating (*k* = 2), absolute agreement, 2-way mixed-effects model [25] In case of dissonance between the assessors, a third assessor evaluated the staining and agreement was reached by discussion.

## Results

A total of 86 NSND were included in this study (Table 1). These patients had a median age of 77.7 years (interquartile range = 14.9 years) and were mainly women (83 %) without regional or distant metastases (69 % and 95 %, respectively). The eight patients who were treated in a palliative setting received either no treatment or palliative radiotherapy. All 78 patients who were treated with curative intend received surgery, 27 of which with postoperative radiotherapy. Twenty-two patients (26 %) had recurrent disease within 5 years. An excellent inter-observer agreement for PD-L1 evaluations (ICC = 0.92) and a good agreement for TILs scoring was achieved (ICC = 0.87).

**Table 1:** Clinical characteristics of oral squamous cell carcinoma in non-smokers and non-drinkers.

Clinical characteristics		Total (*n* = 86)	
Age (years)	Median (interquartile range)	77.7	(14.9)
		n	(%)
Sex	Female	71	(83)
	Male	15	(17)
T-stage	1	23	(27)
	2	30	(35)
	3	7	(8.1)
	4	26	(30)
N-stage	0	59	(69)
	1	15	(17)
	2	10	(12)
	3	2	(2.3)
M-stage	0	82	(95)
	1	4	(4.7)
Recurrence	Yes	22	(26)
	No	64	(74)
Hospital	Utrecht	62	(72)
	Maastricht	24	(28)
Treatment	None	2	(2.3)
	Radiotherapy	6	(7.0)
	Surgery	51	(59)
	Surgery with postoperative radiotherapy	27	(31)
Treatment intent	Curative	78	(91)
	Palliative	8	(9.3)

Eighty-eight percent (76/86) of the OSCC were PD-L1-positive with a CPS ≥1, of which 41 tumors had a CPS ≥20. Tumors with a CPS ≥20 had significantly higher infiltrates of PD-1, CD45, CD8, CD4, and CD3-positive TILs compared to tumors with a CPS <1, and significantly more PD-L2 expression, higher infiltrates of CD45, CD8, and CD3-positive TILs, and a higher CD8/FoxP3 ratio compared to tumors with a CPS 1–19 (Table 2). Forty-three percent (35/81) of the tumors were both PD-L1-positive and had a high CD8-positive TIL infiltrate (PD-L1+/TIL+). Besides more PD-L2 expression and more PD-1, CD45, CD8, CD4, CD3, and FoxP3-positive TILs in the Maastricht patients, there were no significant differences in clinical characteristics between patients with PD-L1-positive and PD-L1-negative OSCC, or a high or low immune cell infiltrate with the analyzed TILs (Supplementary Table 2).

**Table 2:** PD-L1 combined positive score versus PD-L2 tumor proportion score and tumor infiltrating lymphocytes in oral squamous cell carcinoma of non-smokers and non-drinkers.

			Total+		PD-L1 <1		PD-L1 1–19		PD-L1 ≥20		
Marker	Median TILs/mm^2^		n	(%)	n	(%)	n	(%)	n	(%)	p-value
PD-L2	N/A	Positive^(≥1)^	10	(12)	0	(0)	1	(2.9)	9	(23)	**0.015^⁎⁎^**
		Negative	75	(88)	10	(100)	34	(97)	31	(78)	
PD-1	56	High^(>100)^	25	(30)	0	(0)	8	(25)	17	(42)	0.027*
		Low	58	(70)	10	(100)	24	(75)	24	(59)	
CD45	216	High^(>150)^	49	(60)	2	(20)	12	(39)	35	(85)	<0.001^*, **^
		Low	33	(40)	8	(80)	19	(61)	6	(15)	
CD8	121	High^(>150)^	36	(44)	1	(10)	10	(30)	25	(66)	<0.001^*, **^
		Low	45	(56)	9	(90)	23	(70)	13	(34)	
CD4	204	High^(>150)^	48	(59)	2	(20)	18	(54)	28	(72)	0.010*
		Low	34	(42)	8	(80)	15	(46)	11	(28)	
CD3	238	High^(>150)^	54	(64)	2	(20)	14	(42)	38	(93)	<0.001^*, **^
		Low	30	(36)	8	(80)	19	(58)	3	(7.3)	
FoxP3	50	High^(>100)^	27	(33)	2	(22)	13	(38)	12	(32)	0.63
		Low	54	(67)	7	(78)	21	(62)	26	(68)	
CD8/FoxP3 ratio	2.4	High^(≥2.5)^	33	(47)	5	(63)	9	(30)	19	(59)	**0.045^⁎⁎^**
		Low	37	(53)	3	(38)	21	(70)	32	(41)	

+ , Patients were excluded when the TMA showed <10 % tumor cells; N/A, not applicable; ^(≥1)^, cutoff ≥1 %; ^(>100)^, cutoff >100 TILs/mm^2^; ^(>150)^, cutoff 150 TILs/mm^2^; ^(≥2.5)^, cutoff ≥2.5; *, significant difference between PD-L1 <1 and PD-L1 ≥20; ^⁎⁎^, significant difference between PD-L1 1–19 and PD-L1 ≥20.

**Fig. 2:**
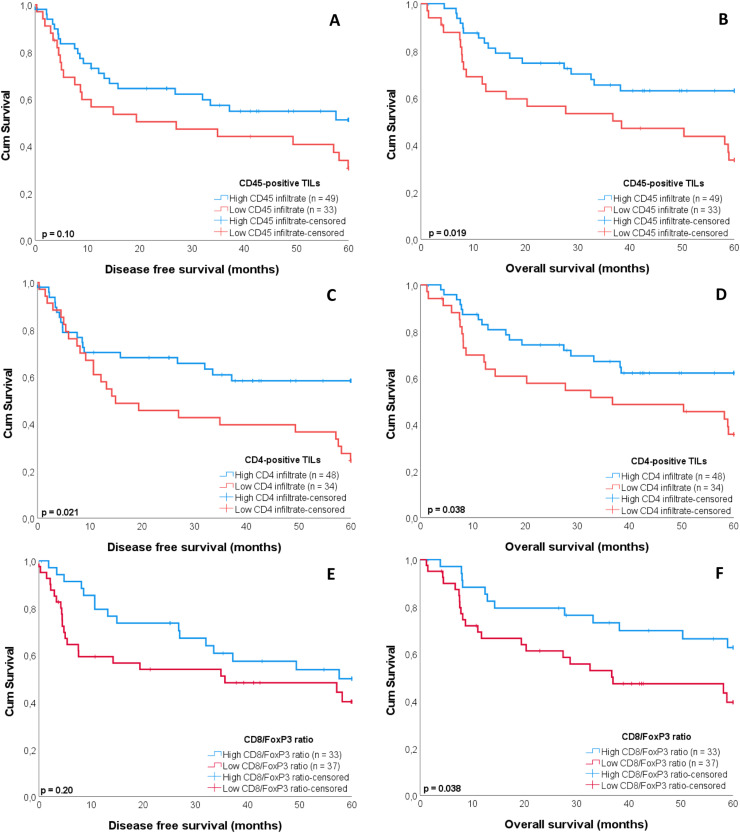
Kaplan-Meier curves estimating survival regarding tumor infiltrating lymphocytes (TILs) positive for CD45 (**A, B**), CD4 (**C, D**), and the CD8/FoxP3 ratio (**E, F**) in oral squamous cell carcinoma of non-smokers and non-drinkers. A high number of CD4-positive TILs showed a significantly better disease free survival (*p* = 0.021) and a high number of CD45-positive TILs, CD4-positive TILs, and a high CD8/FoxP3 ratio a significantly better overall survival than patients with a low number of these TILs (*p* = 0.019, *p* = 0.038, and *p* = 0.038, respectively).

PD-L1 and PD-L2 were no predictors for DFS or OS, regardless of PD-L1 cutoff (≥1 or ≥20). For the TILs, patients with a high number of CD4-positive TILs showed a better DFS than patients with a low number of CD4-positive TILs (*p* = 0.021; Fig. 2). CD4-positive TILs were also retained in the best multivariable model as an independent predictor for DFS (*p* = 0.010, Table 3). Additionally, the N-stage was an independent predictor for DFS (omnibus test of model coefficients: χ^2^ = 20.1 and p < 0.001). Patients with a high number of CD45-positive TILs, CD4-positive TILs, and a high CD8/FoxP3 ratio showed a better OS than patients with a low infiltrate with these TILs (*p* = 0.019, *p* = 0.038, and *p* = 0.038, respectively; Fig. 2). CD4-positive TILs were also retained in the best multivariable model as an independent predictor for OS (*p* = 0.002) and the CD8/FoxP3 ratio was a near significant predictor (*p* = 0.050, Table 4). Additionally, the N-stage was an independent predictor for OS (omnibus test of model coefficients: χ^2^ = 52.7 and p < 0.001). The other TIL markers did not show a significant difference in survival (Supplementary figure 2).

**Table 3:** Multivariable analysis of predictors for disease free survival in oral squamous cell carcinoma of non-smokers and non-drinkers.

Parameters	Coefficient (β)	Standard error	Wald	p-value
N-stage (reference: N0)				
N1	1.1	0.39	8.3	**0.004**
N2	1.1	0.58	3.8	0.050
N3	3.1	1.1	7.8	**0.005**
CD4-positive TILs	0.92	0.36	6.6	**0.010**

TILs, tumor infiltrating lymphocytes.

**Table 4:** Multivariable analysis of predictors for overall survival in oral squamous cell carcinoma of non-smokers and non-drinkers.

Parameters	Coefficient (β)	Standard error	Wald	p-value
N-stage (reference: N0)				
N1	1.6	0.45	12	**<0.001**
N2	1.9	0.72	7.0	**0.008**
N3	6.1	1.5	16	**<0.001**
CD4-positive TILs	1.4	0.45	9.6	**0.002**
CD8/FoxP3 ratio	0.82	0.42	3.8	0.050

TILs, tumor infiltrating lymphocytes.

## Discussion

In this study of NSND, a large number (88 %) of OSCC showed PD-L1 expression (CPS ≥1). PD-L1 CPS ≥20 OSCC had significantly more PD-L2 expression, higher TIL infiltrates and a higher CD8/FoxP3 ratio compared to tumors with a CPS 1–19 or <1. Patients with high numbers of CD4-positive TILs showed a better DFS and OS than patients with low numbers of CD4-positive TILs. Additionally, patients with high numbers of CD45-positive TILs and a high CD8/FoxP3 ratio showed a better OS. High numbers of CD4-positive TILs were an independent predictor for a better DFS and OS, and a high CD8/FoxP3 ratio was a near significant predictor for a better OS. Over 40 % of OSCC were PD-L1+/TIL+.

The PD-L1 expression found in OSCC of NSND in the current study was higher than most of the included studies in a meta-analysis by Tang et al., where the correlation between PD-L1 expression and patient outcomes was evaluated (including smokers and drinkers). They presented one study with 87 % PD-L1-positivity in OSCC, while all other reported studies on OSCC showed 7.3 % - 54.2 % PD-L1 expression [26,27] This is considerably lower than the 88 % found in the current study. An explanation for the higher PD-L1 expression in the current study could be the inclusion of only OSCC from NSND, where differences in the microenvironment are suggested to be more prominent compared to tumors of smokers and drinkers [7,8] Moreover, there are different CPS and TPS cutoff values for PD-L1-positivity used in the literature which are usually higher than the CPS cutoff of ≥1 for PD-L1 in HNSCC that is presented in the current study, therefore resulting in less patients with PD-L1-positive scores [17,27,28] Additionally, there are different PD-L1 staining assays, of which the standardized SP263 assay (as used in this study) and 22C3 PharmaDx are the two that are predominantly being used in diagnostics in The Netherlands [10,17] Although some studies conclude that there is substantial interchangeability between these PD-L1 assays in HNSCC, others report that the SP263 assay produces a more robust staining, resulting in a more frequent CPS ≥1 [17,[29], [30], [31]]

PD-L1 expression was no predictor of DFS or OS in this study, regardless of the CPS cutoff ≥1 or ≥20. This is in line with the outcomes of a systematic review and meta-analysis on the prognostic role of PD-L1 expression in HNSCC, in which in the subgroup analyses of different primary cancer sites no significant prognostic role for PD-L1 was found either [28] Conversely, our results contradict another systematic review and meta-analysis on the prognostic and clinicopathological significance of PD-L1 in just OSCC, in which PD-L1 overexpression was found to be a predictor of DFS and disease specific survival (in addition to significantly more overexpression in NSND) [8] Again, this discrepancy could be explained by the pooling of different PD-L1 cutoffs, since both studies adopted PD-L1-positivity however defined in each original article. Conversely, our study only used the clinically relevant CPS cutoffs of ≥1 and ≥20 because those cutoffs are associated with a favorable response to ICIs [10]

In the current study, PD-L2 did not predict survival. This is in line with a systematic review on immune checkpoints in OSCC where no significant prognostic value was found for PD-L2 [32] All ten PD-L2-positive OSCC in this study were PD-L1-positive too, with nine having a PD-L1 CPS ≥20. This significant association between PD-L1 and PD-L2 expression has been demonstrated before, in addition to a better response to pembrolizumab than non-PD-L2-expressing HNSCC [12]

In the OSCC of this study, a high concentration of CD4-positive TILs significantly predicted both a better DFS and OS. This is in accordance with the systematic review and meta-analysis on the prognostic role of TILs in HNSCC by de Ruiter et al., who reported that in HPV-negative cohorts high CD4-positive TIL numbers were associated with a better OS and locoregional control [13] Though it contradicts with another meta-analysis, in which CD4-positive TILs were reported to be associated with a lower risk of death in OPSCC, but not in OSCC [33] Moreover, both studies reported on the favorable survival associated with high concentrations of CD8-positive TILs, which was not found in our study. This could be explained by both meta-analyses evaluating many OPSCC, which are associated with both more CD8-positive TILs and a better survival. In our study, patients with a high concentration of CD45-positive TILs in their tumors showed a better OS, although CD45 was not retained in the multivariable model as an independent predictor for OS. CD45 has been analyzed less frequently than other TIL markers in HNSCC, but our results are in accordance with another study that suggests that CD45 expression could be a potential marker for tumor outcome in HNSCC patients [34] The finding that a high CD8/FoxP3 ratio was a near significant predictor of OS in OSCC also matches previous reports [23,[35], [36], [37]]

Our results are relevant in the light of the increasing use of ICIs in HNSCC, which might be beneficial not only for palliative care of patients with recurrent or metastatic HNSCC, but also as neoadjuvant treatment. In this study, 43 % of OSCC had a high CD8-positive TIL infiltrate combined with a PD-L1-positive tumor, matching an “adaptive immune resistance” tumor microenvironment [15] Therefore, these patients could in theory be interesting candidates for neoadjuvant ICIs. Nevertheless, even in the tumors with PD-L1+/TIL+ there are non-responders to ICIs [38] Thus, further research is required to evaluate if the combination between PD-L1 and TILs are accurate predictors of a response to ICIs in NSND.

One of the limitations of this study was that some of the FFPE material had >25 years of storage, resulting in slight differences in staining intensity between the tumors with a longer and shorter storage time. Indeed, the tumors from the older Utrecht cohort showed overall less TILs than the tumors from the newer Maastricht cohort. However, the TMA blocks were freshly sectioned before IHC, all positive controls were adequate, and there was no difference in PD-L1 expression observed between the cohorts. Also, in a previous study IHC staining for p53 and pRb did not show significant differences in expression between both cohorts [1] Therefore, the most plausible explanation might just be differences in the number of TILs in both cohorts as shown by the pan-leukocyte marker CD45. This marker acts as an internal positive control, as it has to stain all leukocytes. This was the case in the current study and no lymphocytes without staining could be identified within the TMA that was stained for CD45, indicating that the immune cell populations were correctly represented. Because the other TIL markers had a strong positive correlation to CD45, the difference in TILs between the cohorts is also reflected by these markers. Secondly, this study used TMAs which only comprises a part of the original tumor. Though, three tumor cores were taken per patient to take into account some heterogeneity within the tumor [39,40] Furthermore, some sections further into the TMA block did not show tumor cells anymore in some TMA cores, resulting in not all TIL markers being available for all patients. Because we present a well-defined cohort of NSND (for example with exclusion of patients with “sporadic” alcohol consumption), the cohort was somewhat limited in size so selection was not based on availability of all TIL markers per patient. Although, encompassing 86 cases it still is one of the largest NSND cohorts in the literature [3] Finally, the tumors were staged according to the AJCC seventh edition instead of the current eight edition. At the time of the tumor samples collection the seventh edition was used and we expected more inaccuracies when restaging some of the 25-year-old tumors than keeping the seventh edition staging, because data on extra-nodal extension might not be available in 25-year-old pathology reports. Regardless, our conclusions regarding PD-L1 expression or CD4-positive TILs related survival would not change if the tumors were staged differently.

## Conclusion

In NSND, a large number of OSCC show PD-L1 expression (CPS ≥1). CD4 was a significant predictor of DFS and OS, in addition to the CD8/FoxP3 ratio being a near significant predictor for OS. Since there is frequent PD-L1 expression in tumors with a high infiltrate of CD8-positive TILs (constituting an “adaptive immune resistance” tumor microenvironment), NSND should in theory be interesting candidates for treatment with ICIs.

## CRediT authorship contribution statement

**FJ Mulder:** Writing – original draft, Methodology, Formal analysis, Data curation. **EJ de Ruiter:** Writing – review & editing, Data curation. **TFB Gielgens:** Writing – original draft, Data curation. **F Farshadpour:** Writing – review & editing, Conceptualization. **R de Bree:** Writing – review & editing, Data curation. **MFCM van den Hout:** Writing – review & editing, Validation. **B Kremer:** Writing – review & editing, Supervision, Conceptualization. **SM Willems:** Writing – review & editing, Supervision, Methodology. **EJM Speel:** Writing – review & editing, Supervision, Methodology, Conceptualization.

## Declaration of competing interest

The authors declare the following financial interests/personal relationships which may be considered as potential competing interests:

Bernd Kremer reports a relationship with Maastricht University Medical Center that includes: funding grants. Ernst-Jan Speel reports a relationship with Maastricht University Medical Center that includes: funding grants. Stefan Willems reports a relationship with University Medical Center Groningen that includes: funding grants. If there are other authors, they declare that they have no known competing financial interests or personal relationships that could have appeared to influence the work reported in this paper.

## Appendix Supplementary materials

Image, application 1
