# Supplementary material for: Frequent PD-L1 expression in oral squamous cell carcinoma of non-smokers and non-drinkers, and association of tumor infiltrating lymphocytes with favorable prognosis
Source: Transl Oncol. 2025 Mar 15;55:102357. doi: 10.1016/j.tranon.2025.102357 (PMC11957585; doi:10.1016/j.tranon.2025.102357)
Supplement: Supplementary file 1 [file mmc1.docx]

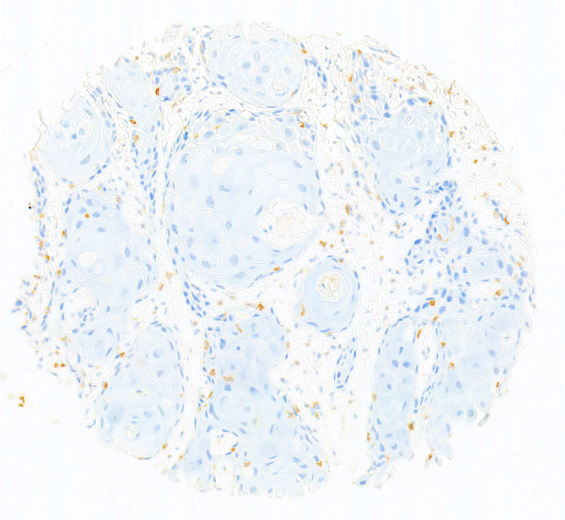

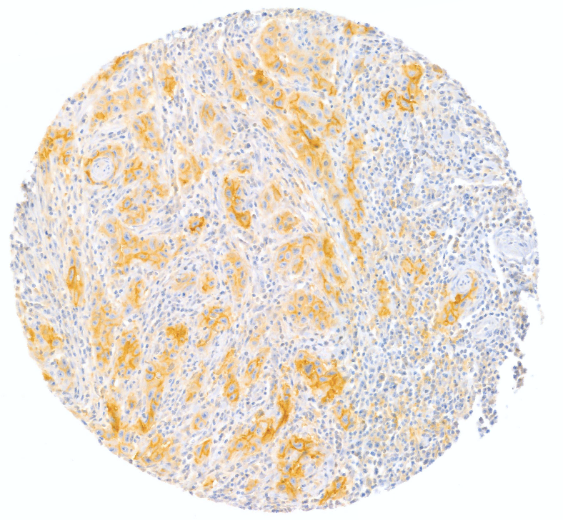

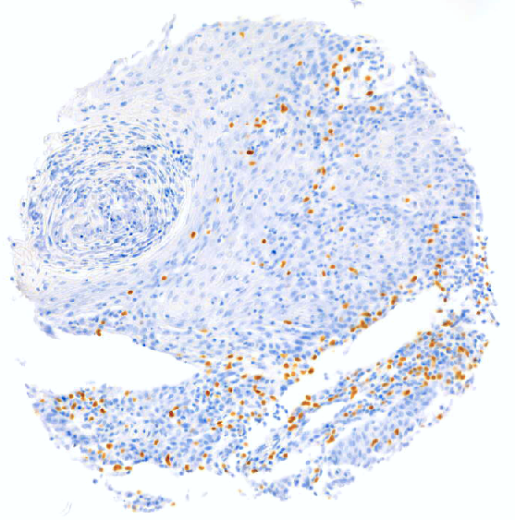

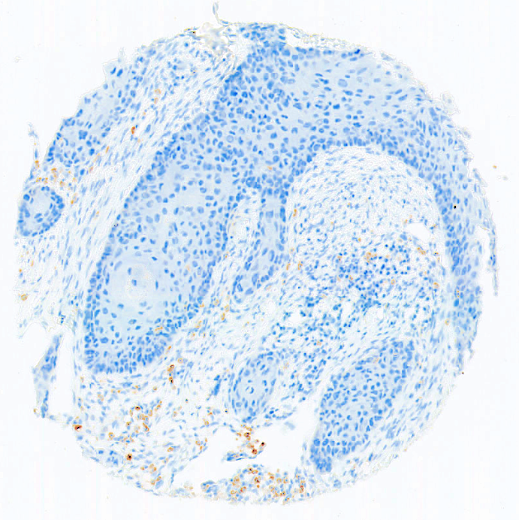

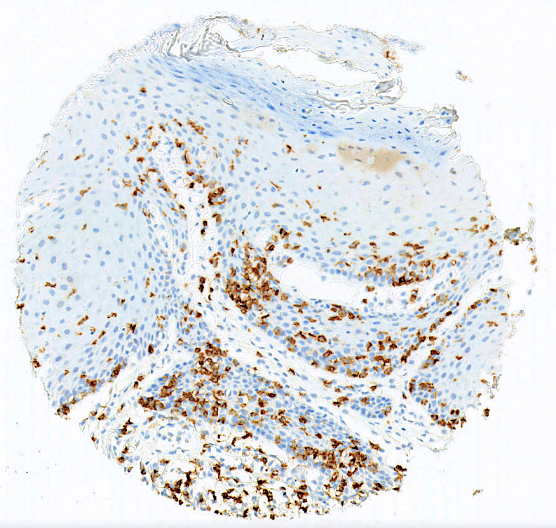

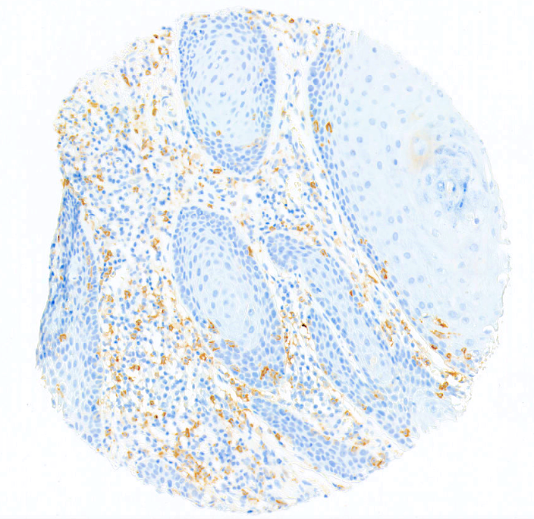


**B**

**A**

🡪

🡪

🡪

🡪

**Supplementary figure 1**. Example of a tissue micro array core with staining for PD-L2 (**A**), PD-1 (**B**), CD8 (**C**), CD4 (**D**), CD3 (**E**), and FoxP3 (**F**). A tumor infiltrating lymphocyte positive for each staining is marked with 🡪 in images **B**-**F**. Images were digitally evaluated at 200x magnification as shown.

**F**

**E**

**D**

**C**

🡪


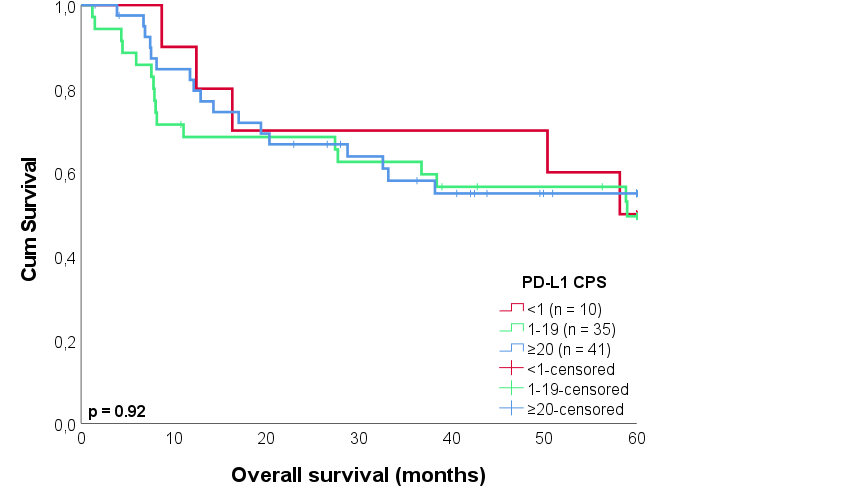

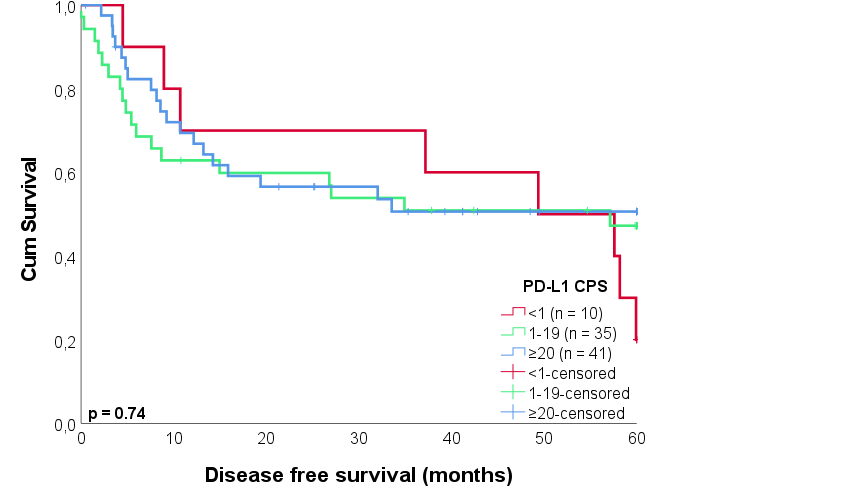

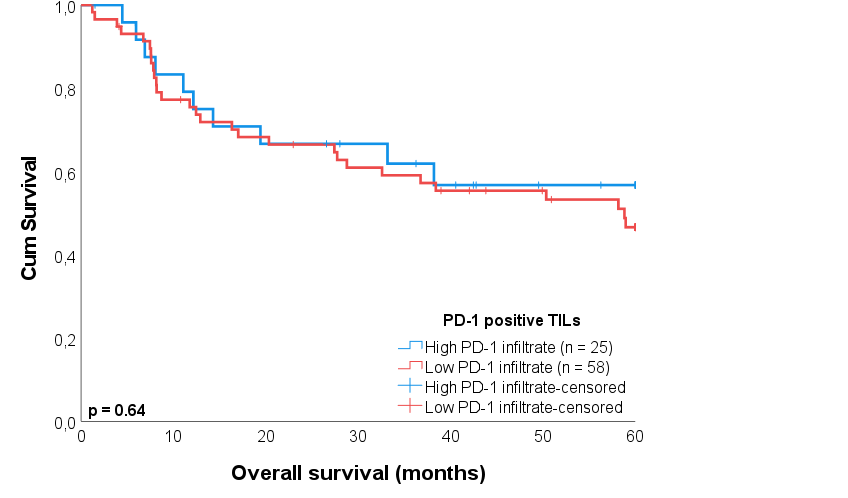

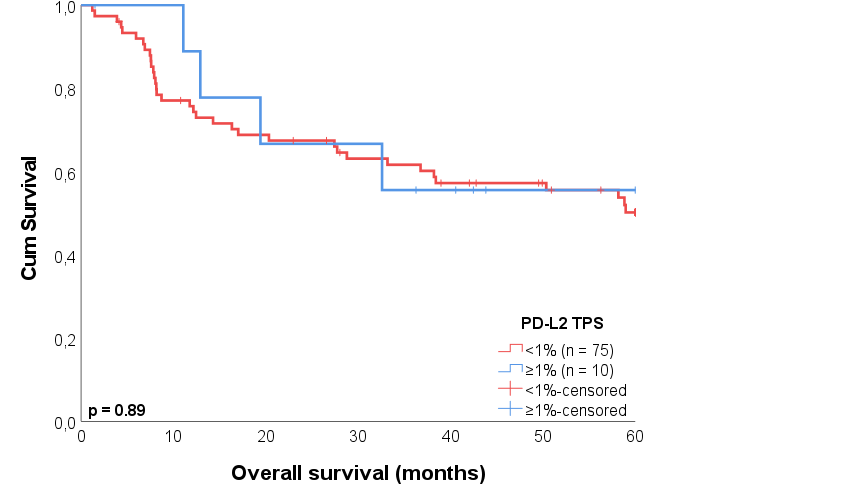

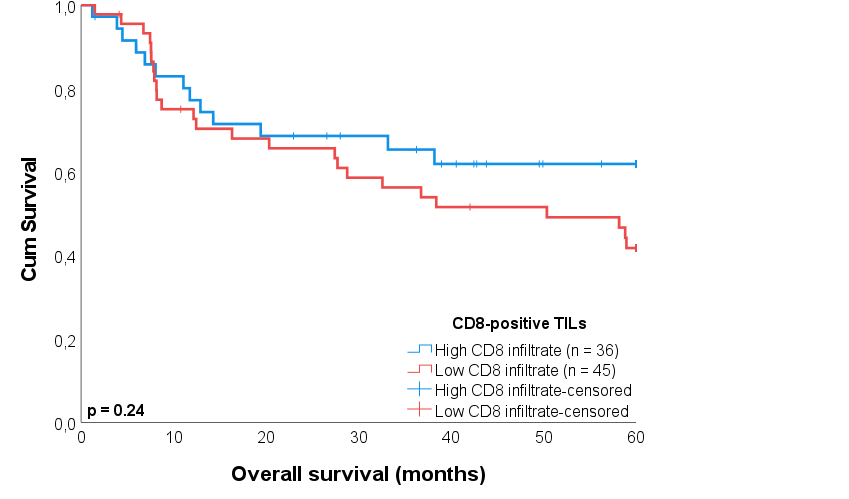

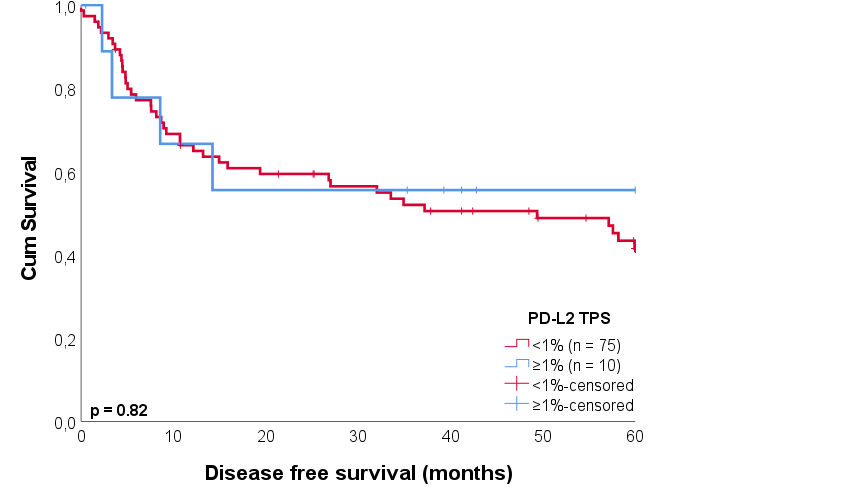


**B**

**A**

**F**

**D**

**E**

**C**


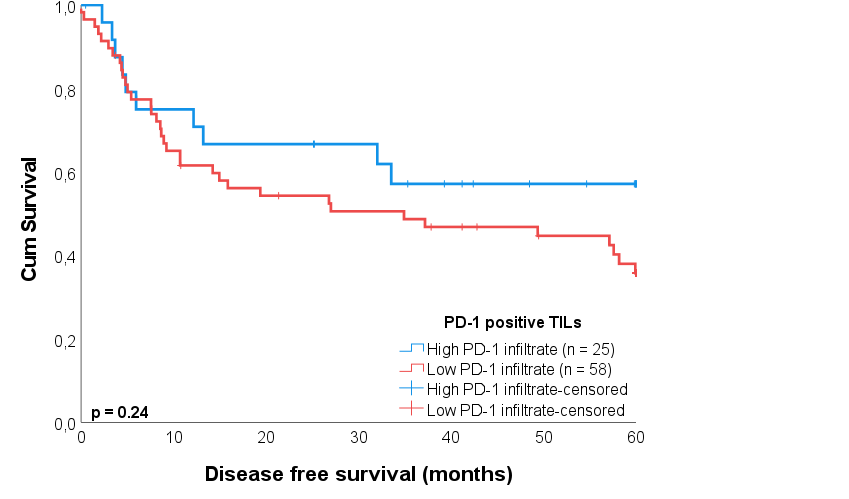


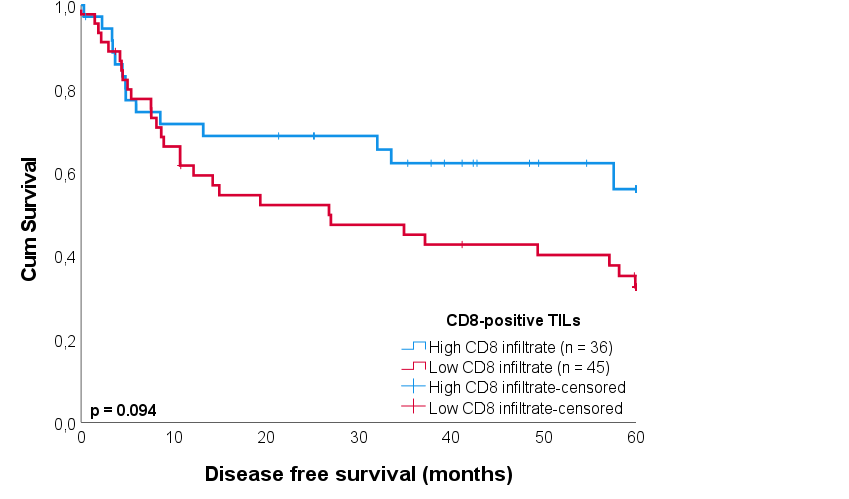


**H**

**G**


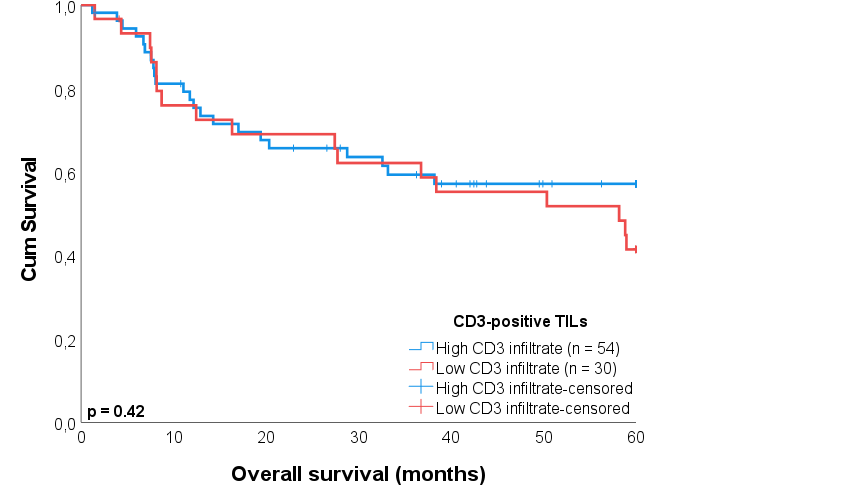

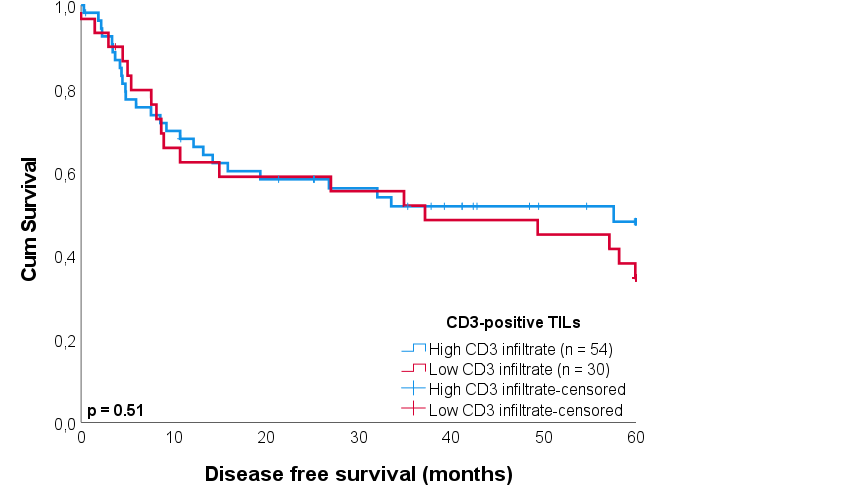


**K**

**I**

**J**


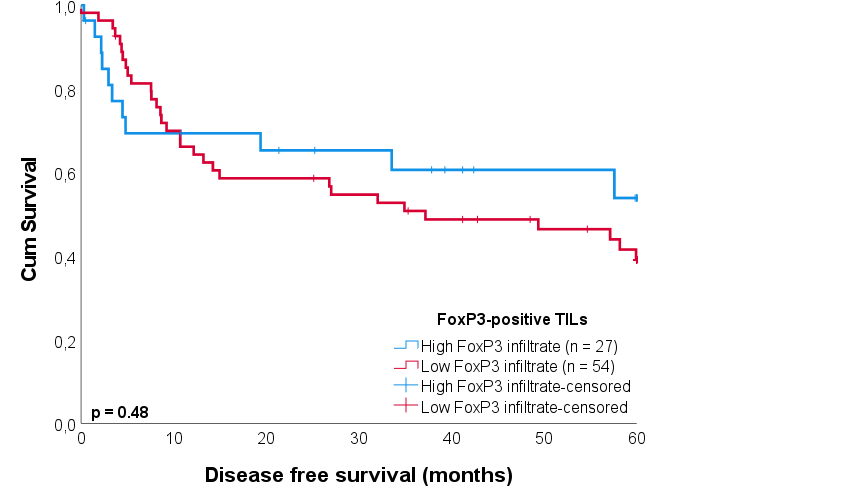

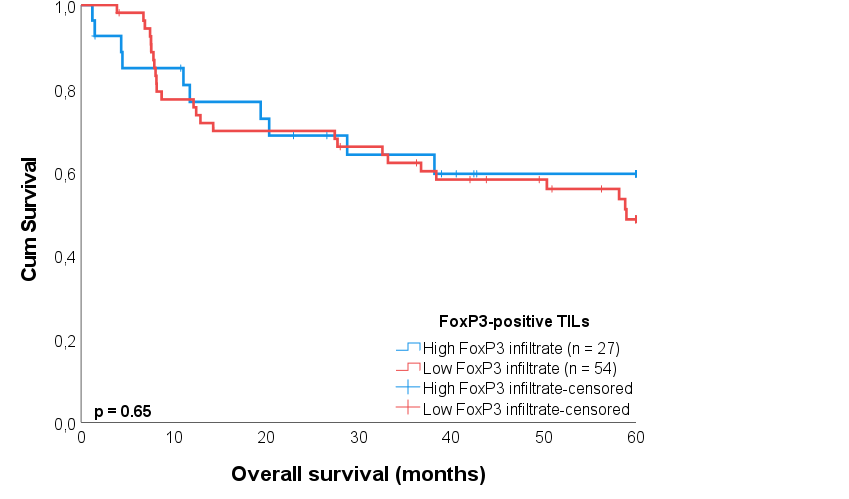


**L**

**Supplementary figure 2**. Kaplan-Meier curves estimating survival regarding PD-L1 combined positive score (CPS; **A, B**), PD-L2 tumor proportion score (TPS; **C, D**), and tumor infiltrating lymphocytes (TILs) positive for PD-1 (**E, F**), CD8 (**G, H**), CD3 (**I, J**), and FoxP3 (**K, L**) in oral squamous cell carcinoma of non-smokers and non-drinkers. These markers did not show a significant difference in disease free survival or overall survival.

| **Supplementary table 1. Immunohistochemistry primary antibodies and evaluation criteria** | | | | | | | |
| --- | --- | --- | --- | --- | --- | --- | --- |
| *Antibody characteristics* | | *Source* | *Clone* | *Dilution* | *Retrieval* | *Localization* | *Evaluation criteria* |
| *Antibody* | *Company* |  |  |  |  |  | *Cutoff* |
| PD-L1 | Roche Diagnostics | Monoclonal, rabbit | SP263 | Ready-to-use | High pH buffer (pH 9.0) | Cell membrane | ≥1 |
| PD-L2 | Cell Signaling | Monoclonal, rabbit | D7U8C | 1:100 | High pH buffer (pH 9.0) | Cell membrane | ≥1% |
| PD-1 | Cell Marque | Monoclonal, mouse | MRQ22 | 1:50 | Low pH buffer (pH 6.0) | Cytoplasm | 100 TILs/mm² |
| CD45 | Dako Omnis | Monoclonal, mouse | 2B11 + PD7/26 | Ready-to-use | Low pH buffer (pH 6.0) | Cell membrane | 150 TILs/mm² |
| CD8 | Dako Omnis | Monoclonal, mouse | C8/144B | Ready-to-use | High pH buffer (pH 9.0) | Cell membrane and cytoplasm | 150 TILs/mm² |
| CD4 | Dako Omnis | Monoclonal, mouse | 4B12 | Ready-to-use | High pH buffer (pH 9.0) | Cell membrane | 150 TILs/mm² |
| CD3 | Dako Omnis | Polyclonal, rabbit | - | Ready-to-use | High pH buffer (pH 9.0) | Cell membrane and cytoplasm | 150 TILs/mm² |
| FoxP3 | Abcam | Monoclonal, mouse | 236A/E7 | 1:100 | High pH buffer (pH 9.0) | Cell membrane and cytoplasm | 100 TILs/mm² |
| TILs, tumor infiltrating lymphocytes | | | | | | | |

| **Supplementary table 2. Comparison between clinical characteristics and PD-L1 combined positive score (CPS), PD-L2 tumor proportion score (TPS) and tumor infiltrating lymphocyte markers in oral squamous cell carcinoma of non-smokers and non-drinkers** | | | | | | | | | | | | | | | | | | | | | | |
| --- | --- | --- | --- | --- | --- | --- | --- | --- | --- | --- | --- | --- | --- | --- | --- | --- | --- | --- | --- | --- | --- | --- |
| *Clinical characteristics* | |  | | *PD-L1 CPS ≥1* | | *PD-L1 CPS <1* | |  |  | | *PD-L1 CPS ≥20* | | *PD-L1 CPS <20* | |  |  |  | *PD-L2 TPS ≥1%* | | *PD-L2 TPS <1%* | |  |
|  |  | *Total (n = 86)* | | *(n = 76)* | | *(n = 10)* | | *p-value* | *Total (n = 86)* | | *(n = 41)* | | *(n = 45)* | | *p-value* | *Total (n = 85)^+^* | | *(n = 10)* | | *(n = 75)* | | *p-value* |
| Age in years | Median (interquartile range) | 77.7 | (14.9) | 75.9 | (14.2) | 79.5 | (7.7) | 0.19 | 77.7 | (14.9) | 75.5 | (15.1) | 77.8 | (14.2) | 0.96 | 77.8 | (14.2) | 76.6 | (16.6) | 77.8 | (13.6) | 0.63 |
|  |  | *n* | *(%)* | *n* | *(%)* | *n* | *(%)* |  | *n* | *(%)* | *n* | *(%)* | *n* | *(%)* |  | *n* | *(%)* | *n* | *(%)* | *n* | *(%)* |  |
| Sex | Female | 71 | (83) | 64 | (84) | 7 | (70) | 0.37 | 71 | (83) | 36 | (88) | 35 | (78) | 0.22 | 70 | (82) | 9 | (90) | 61 | (81) | 0.68 |
|  | Male | 15 | (17) | 12 | (16) | 3 | (30) |  | 15 | (17) | 5 | (12) | 10 | (22) |  | 15 | (18) | 1 | (10) | 14 | (19) |  |
| T-stage | 1 | 23 | (27) | 19 | (25) | 4 | (40) | 0.53 | 23 | (27) | 10 | (24) | 13 | (29) | 0.36 | 23 | (27) | 3 | (30) | 20 | (27) | 1.0 |
|  | 2 | 30 | (35) | 28 | (37) | 2 | (20) |  | 30 | (35) | 18 | (44) | 12 | (27) |  | 29 | (34) | 3 | (30) | 26 | (35) |  |
|  | 3 | 7 | (8.1) | 7 | (9.2) | 0 | (0) |  | 7 | (8.1) | 2 | (4.9) | 5 | (11) |  | 7 | (8.2) | 1 | (10) | 6 | (8.0) |  |
|  | 4 | 26 | (30) | 22 | (29) | 4 | (40) |  | 26 | (30) | 11 | (27) | 15 | (33) |  | 26 | (31) | 3 | (30) | 23 | (31) |  |
| N-stage | 0 | 59 | (69) | 52 | (68) | 7 | (70) | 0.50 | 59 | (69) | 28 | (68) | 31 | (69) | 1.0 | 58 | (68) | 6 | (60) | 52 | (69) | 0.68 |
|  | 1 | 15 | (17) | 12 | (16) | 3 | (30) |  | 15 | (17) | 7 | (17) | 8 | (18) |  | 15 | (18) | 2 | (20) | 13 | (17) |  |
|  | 2 | 10 | (12) | 10 | (13) | 0 | (0) |  | 10 | (12) | 5 | (12) | 5 | (11) |  | 10 | (12) | 2 | (20) | 8 | (11) |  |
|  | 3 | 2 | (2.3) | 2 | (2.6) | 0 | (0) |  | 2 | (2.3) | 1 | (2.4) | 1 | (2.2) |  | 2 | (2.4) | 0 | (0) | 2 | (2.7) |  |
| M-stage | 0 | 82 | (95) | 72 | (95) | 10 | (100) | 1.0 | 82 | (95) | 38 | (93) | 44 | (98) | 0.34 | 81 | (95) | 8 | (80) | 73 | (97) | 0.066 |
|  | 1 | 4 | (4.7) | 4 | (5.3) | 0 | (0) |  | 4 | (4.7) | 3 | (7.30 | 1 | (2.2) |  | 4 | (4.7) | 2 | (20) | 2 | (2.8) |  |
| Recurrence | Yes | 22 | (26) | 17 | (22) | 5 | (50) | 0.12 | 22 | (26) | 10 | (24) | 12 | (27) | 1.0 | 22 | (26) | 4 | (40) | 18 | (24) | 0.28 |
|  | No | 64 | (74) | 59 | (78) | 5 | (50) |  | 64 | (74) | 31 | (76) | 33 | (73) |  | 63 | (74) | 6 | (60) | 57 | (76) |  |
| Hospital | Utrecht | 62 | (72) | 55 | (72) | 7 | (70) | 1.0 | 62 | (72) | 28 | (68) | 34 | (76) | 0.48 | 61 | (72) | 2 | (20) | 59 | (79) | **<0.001** |
|  | Maastricht | 24 | (28) | 21 | (28) | 3 | (30) |  | 24 | (28) | 13 | (32) | 11 | (24) |  | 24 | (28) | 8 | (80) | 16 | (21) |  |
| Treatment | None | 2 | (2.3) | 2 | (2.6) | 0 | (0) | 0.38 | 2 | (2.3) | 0 | (0) | 2 | (4.4) | 0.31 | 2 | (2.4) | 0 | (0) | 2 | (2.7) | 1.0 |
|  | Radiotherapy | 6 | (7) | 4 | (5.3) | 2 | (20) |  | 6 | (7.0) | 2 | (4.9) | 4 | (8.9) |  | 6 | (7.1) | 0 | (0) | 6 | (8.0) |  |
|  | Surgery | 51 | (59) | 46 | (61) | 5 | (50) |  | 51 | (59) | 28 | (68) | 23 | (51) |  | 50 | (59) | 7 | (70) | 43 | (57) |  |
|  | Surgery with postoperative radiotherapy | 27 | (31) | 24 | (32) | 3 | (30) |  | 27 | (31) | 11 | (27) | 16 | (36) |  | 27 | (32) | 3 | (30) | 24 | (32) |  |
| Treatment intent | Curative | 78 | (91) | 70 | (92) | 8 | (80) | 0.23 | 78 | (91) | 39 | (95) | 39 | (87) | 0.27 | 77 | (91) | 10 | (100) | 67 | (89) | 0.59 |
|  | Palliative | 8 | (9.3) | 6 | (7.9) | 2 | (20) |  | 8 | (9.3) | 2 | (4.9) | 6 | (13) |  | 8 | (9.4) | 0 | (0) | 8 | (11) |  |
| ^+^, Patients were excluded when the TMA showed <10% tumor cells | | | | | | | | | | | | | | | | | | | | | | |

| **Supplementary table 2. Comparison between clinical characteristics and PD-L1 combined positive score (CPS), PD-L2 tumor proportion score (TPS) and tumor infiltrating lymphocyte markers in oral squamous cell carcinoma of non-smokers and non-drinkers** | | | | | | | | | | | | | | | | | | | | | | |
| --- | --- | --- | --- | --- | --- | --- | --- | --- | --- | --- | --- | --- | --- | --- | --- | --- | --- | --- | --- | --- | --- | --- |
| *Clinical characteristics* | |  |  | *PD-1 high infiltrate^(>100)^* | | *PD-1 low infiltrate* | |  |  |  | *CD45 high infiltrate^(>150)^* | | *CD45 low infiltrate* | |  |  |  | *CD8 high infiltrate^(>150)^* | | *CD8 low infiltrate* | |  |
|  |  | *Total (n = 83)^+^* | | *(n = 25)* | | *(n = 58)* | | *p-value* | *Total (n = 82)^+^* | | *(n = 49)* | | *(n = 33)* | | *p-value* | *Total (n = 81)^+^* | | *(n = 36)* | | *(n = 45)* | | *p-value* |
| Age in years | Median (interquartile range) | 77.6 | (14.8) | 79.8 | (14.5) | 76.2 | (14.7) | 0.69 | 77.9 | (15.0) | 78.8 | (14.2) | 77.6 | (18.2) | 0.87 | 77.8 | (14.9) | 76.4 | (16.7) | 77.9 | (15.1) | 0.21 |
|  |  | *n* | *(%)* | *n* | *(%)* | *n* | *(%)* |  | *n* | *(%)* | *n* | *(%)* | *n* | *(%)* |  | *n* | *(%)* | *n* | *(%)* | *n* | *(%)* |  |
| Sex | Female | 70 | (84) | 22 | (88) | 48 | (83) | 0.75 | 67 | (82) | 43 | (88) | 24 | (73) | 0.14 | 67 | (83) | 30 | (83) | 37 | (82) | 1.0 |
|  | Male | 13 | (16) | 3 | (12) | 10 | (17) |  | 15 | (18) | 6 | (12) | 9 | (27) |  | 14 | (17) | 6 | (17) | 8 | (18) |  |
| T-stage | 1 | 22 | (27) | 6 | (24) | 16 | (28) | 0.31 | 21 | (26) | 14 | (29) | 7 | (21) | 0.54 | 22 | (27) | 9 | (25) | 13 | (29) | 0.29 |
|  | 2 | 29 | (35) | 11 | (44) | 18 | (31) |  | 29 | (35) | 19 | (39) | 10 | (30) |  | 28 | (35) | 15 | (42) | 13 | (29) |  |
|  | 3 | 6 | (7.2) | 3 | (12) | 3 | (5.2) |  | 7 | (8.5) | 3 | (6.1) | 4 | (12) |  | 6 | (7.4) | 4 | (11) | 2 | (4.4) |  |
|  | 4 | 26 | (31) | 5 | (20) | 21 | (36) |  | 25 | (31) | 13 | (27) | 12 | (36) |  | 25 | (31) | 8 | (22) | 17 | (38) |  |
| N-stage | 0 | 57 | (69) | 16 | (64) | 41 | (71) | 0.22 | 56 | (68) | 35 | (71) | 21 | (64) | 0.36 | 55 | (68) | 22 | (61) | 33 | (73) | 0.11 |
|  | 1 | 15 | (18) | 3 | (12) | 12 | (21) |  | 15 | (18) | 7 | (14) | 8 | (24) |  | 14 | (17) | 5 | (14) | 9 | (20) |  |
|  | 2 | 9 | (11) | 5 | (20) | 4 | (6.9) |  | 10 | (12) | 7 | (14) | 3 | (9.1) |  | 10 | (12) | 7 | (19) | 3 | (6.7) |  |
|  | 3 | 2 | (2.4) | 1 | (4.0) | 1 | (1.7) |  | 1 | (1.2) | 0 | (0) | 1 | (3.0) |  | 2 | (2.5) | 2 | (5.6) | 0 | (0) |  |
| M-stage | 0 | 79 | (95) | 24 | (96) | 55 | (95) | 1.0 | 78 | (95) | 46 | (94) | 32 | (97) | 0.65 | 77 | (95) | 34 | (94) | 43 | (96) | 1.0 |
|  | 1 | 4 | (4.8) | 1 | (4.0) | 3 | (5.2) |  | 4 | (4.9) | 3 | (6.1) | 1 | (3.0) |  | 4 | (4.9) | 2 | (5.6) | 2 | (4.4) |  |
| Recurrence | Yes | 22 | (27) | 4 | (16) | 18 | (31) | 0.19 | 22 | (27) | 12 | (25) | 10 | (30) | 0.62 | 21 | (26) | 7 | (19) | 14 | (31) | 0.31 |
|  | No | 61 | (74) | 21 | (84) | 40 | (69) |  | 60 | (73) | 37 | (76) | 23 | (70) |  | 60 | (74) | 29 | (81) | 31 | (69) |  |
| Hospital | Utrecht | 59 | (71) | 10 | (40) | 49 | (85) | **<0.001** | 58 | (71) | 28 | (57) | 30 | (91) | **0.001** | 57 | (70) | 18 | (50) | 39 | (87) | **<0.001** |
|  | Maastricht | 24 | (29) | 15 | (60) | 9 | (16) |  | 24 | (29) | 21 | (43) | 3 | (9.1) |  | 24 | (30) | 18 | (50) | 6 | (13) |  |
| Treatment | None | 2 | (2.4) | 1 | (4.0) | 1 | (1.7) | 0.55 | 2 | (2.4) | 1 | (2.0) | 1 | (3.0) | 0.35 | 2 | (2.5) | 1 | (2.8) | 1 | (2.2) | 0.45 |
|  | Radiotherapy | 6 | (7.2) | 2 | (8.0) | 4 | (6.9) |  | 5 | (6.1) | 2 | (4.1) | 3 | (9.1) |  | 6 | (7.4) | 2 | (5.6) | 4 | (8.9) |  |
|  | Surgery | 51 | (61) | 17 | (68) | 34 | (59) |  | 49 | (60) | 33 | (67) | 16 | (49) |  | 49 | (61) | 25 | (69) | 24 | (53) |  |
|  | Surgery with postoperative radiotherapy | 24 | (29) | 5 | (20) | 19 | (33) |  | 26 | (32) | 13 | (27) | 13 | (39) |  | 24 | (30) | 8 | (22) | 16 | (36) |  |
| Treatment intent | Curative | 75 | (90) | 22 | (88) | 53 | (91) | 0.69 | 75 | (92) | 46 | (94) | 29 | (88) | 0.43 | 73 | (90) | 33 | (92) | 40 | (89) | 0.73 |
|  | Palliative | 8 | (9.6) | 3 | (12) | 5 | (8.6) |  | 7 | (8.5) | 3 | (6.1) | 4 | (12) |  | 8 | (9.9) | 3 | (8.3) | 5 | (11) |  |
| ^+^, Patients were excluded when the TMA showed <10% tumor cells; ^(>100)^, >100 TILs/mm^2^; ^(>150)^, >150 TILs/mm^2^ | | | | | | | | | | | | | | | | | | | | | | |

| **Supplementary table 2. Comparison between clinical characteristics and PD-L1 combined positive score (CPS), PD-L2 tumor proportion score (TPS) and tumor infiltrating lymphocyte markers in oral squamous cell carcinoma of non-smokers and non-drinkers** | | | | | | | | | | | | | | | | | | | | | | |
| --- | --- | --- | --- | --- | --- | --- | --- | --- | --- | --- | --- | --- | --- | --- | --- | --- | --- | --- | --- | --- | --- | --- |
|  |  |  |  |  |  |  |  |  |  |  |  |  |  |  |  |  |  |  |  |  |  |  |
| *Clinical characteristics* | |  |  | *CD4 high infiltrate^(>150)^* | | *CD4 low infiltrate* | |  |  |  | *CD3 high infiltrate^(>150)^* | | *CD3 low infiltrate* | |  |  |  | *FoxP3 high infiltrate^(>100)^* | | *FoxP3 low infiltrate* | |  |
|  |  | *Total (n = 82)^+^* | | *(n = 48)* | | *(n = 34)* | | *p-value* | *Total (n = 84)^+^* | | *(n = 54)* | | *(n = 30)* | | *p-value* | *Total (n = 81)^+^* | | *(n = 27)* | | *(n = 54)* | | *p-value* |
| Age in years | Median (interquartile range) | 76.9 | (14.9) | 75.3 | (17.1) | 77.7 | (12.9) | 0.57 | 76.9 | (14.9) | 76.6 | (14.8) | 77.0 | (15.5) | 0.72 | 77.9 | (15.0) | 78.8 | (11.9) | 77.9 | (16.6) | 0.79 |
|  |  | *n* | *(%)* | *n* | *(%)* | *n* | *(%)* |  | *n* | *(%)* | *n* | *(%)* | *n* | *(%)* |  | *n* | *(%)* | *n* | *(%)* | *n* | *(%)* |  |
| Sex | Female | 68 | (83) | 42 | (88) | 26 | (77) | 0.24 | 70 | (83) | 47 | (87) | 23 | (77) | 0.24 | 66 | (82) | 24 | (89) | 42 | (78) | 0.36 |
|  | Male | 14 | (17) | 6 | (13) | 8 | (24) |  | 14 | (17) | 7 | (13) | 7 | (23) |  | 15 | (19) | 3 | (11) | 12 | (22) |  |
| T-stage | 1 | 22 | (27) | 14 | (29) | 8 | (24) | 0.49 | 22 | (26) | 14 | (26) | 8 | (27) | 0.23 | 21 | (26) | 9 | (33) | 12 | (22) | 0.60 |
|  | 2 | 28 | (34) | 16 | (33) | 12 | (35) |  | 30 | (36) | 23 | (43) | 7 | (23) |  | 28 | (35) | 7 | (26) | 21 | (39) |  |
|  | 3 | 6 | (7.3) | 5 | (10) | 1 | (2.9) |  | 6 | (7.1) | 4 | (7.4) | 2 | (6.7) |  | 7 | (8.6) | 2 | (7.4) | 5 | (9.3) |  |
|  | 4 | 26 | (32) | 13 | (27) | 13 | (38) |  | 26 | (31) | 13 | (24) | 13 | (43) |  | 25 | (31) | 9 | (33) | 16 | (30) |  |
| N-stage | 0 | 56 | (68) | 31 | (65) | 25 | (74) | 0.54 | 57 | (68) | 36 | (67) | 21 | (70) | 0.87 | 56 | (69) | 20 | (74) | 36 | (57) | 0.96 |
|  | 1 | 14 | (17) | 8 | (17) | 6 | (18) |  | 15 | (18) | 9 | (17) | 6 | (20) |  | 14 | (17) | 4 | (15) | 10 | (19) |  |
|  | 2 | 10 | (12) | 8 | (17) | 2 | (5.9) |  | 10 | (12) | 7 | (13) | 3 | (10) |  | 10 | (12) | 3 | (11) | 7 | (13) |  |
|  | 3 | 2 | (2.4) | 1 | (2.1) | 1 | (2.9) |  | 2 | (2.4) | 2 | (3.7) | 0 | (0) |  | 1 | (1.2) | 0 | (0) | 1 | (1.9) |  |
| M-stage | 0 | 78 | (95) | 46 | (96) | 32 | (94) | 1.0 | 80 | (95) | 52 | (96) | 28 | (93) | 0.61 | 77 | (85) | 26 | (96) | 51 | (94) | 1.0 |
|  | 1 | 4 | (4.9) | 2 | (4.2) | 2 | (5.9) |  | 4 | (4.8) | 2 | (3.7) | 2 | (6.7) |  | 4 | (4.9) | 1 | (3.7) | 3 | (5.6) |  |
| Recurrence | Yes | 22 | (27) | 12 | (25) | 10 | (29) | 0.80 | 22 | (26) | 13 | (24) | 9 | (30) | 0.61 | 21 | (26) | 6 | (22) | 15 | (28) | 0.79 |
|  | No | 60 | (73) | 36 | (75) | 24 | (71) |  | 62 | (74) | 41 | (76) | 21 | (70) |  | 60 | (74) | 21 | (78) | 39 | (72) |  |
| Hospital | Utrecht | 58 | (71) | 26 | (54) | 32 | (94) | **<0.001** | 60 | (71) | 33 | (61) | 27 | (90) | **0.005** | 58 | (72) | 15 | (56) | 43 | (80) | **0.036** |
|  | Maastricht | 24 | (29) | 22 | (46) | 2 | (5.9) |  | 24 | (29) | 21 | (39) | 3 | (10) |  | 23 | (28) | 12 | (44) | 11 | (20) |  |
| Treatment | None | 2 | (2.4) | 1 | (2.1) | 1 | (2.9) | 0.10 | 2 | (2.4) | 1 | (1.9) | 1 | (3.3) | 0.13 | 2 | (2.5) | 2 | (7.4) | 0 | (0) | **<0.001** |
|  | Radiotherapy | 6 | (7.3) | 1 | (2.1) | 5 | (15) |  | 6 | (7.1) | 2 | (3.7) | 4 | (13) |  | 5 | (6.2) | 0 | (0) | 5 | (9.3) |  |
|  | Surgery | 50 | (61) | 33 | (69) | 17 | (50) |  | 51 | (61) | 37 | (69) | 14 | (47) |  | 48 | (59) | 22 | (82) | 26 | (48) |  |
|  | Surgery with postoperative radiotherapy | 24 | (29) | 13 | (27) | 11 | (32) |  | 25 | (30) | 14 | (26) | 11 | (37) |  | 26 | (32) | 3 | (11) | 23 | (43) |  |
| Treatment intent | Curative | 74 | (90) | 46 | (96) | 28 | (82) | 0.061 | 76 | (91) | 51 | (94) | 25 | (83) | 0.13 | 74 | (91) | 25 | (93) | 49 | (91) | 1.0 |
|  | Palliative | 8 | (9.8) | 2 | (4.2) | 6 | (18) |  | 8 | (9.5) | 3 | (5.6) | 5 | (17) |  | 7 | (8.6) | 2 | (7.4) | 5 | (9.3) |  |
| ^+^, Patients were excluded when the TMA showed <10% tumor cells; ^(>100)^, >100 TILs/mm^2^; ^(>150)^, >150 TILs/mm^2^ | | | | | | | | | | | | | | | | | | | | | | |

| **Supplementary table 2. Comparison between clinical characteristics and PD-L1 combined positive score (CPS), PD-L2 tumor proportion score (TPS) and tumor infiltrating lymphocyte markers in oral squamous cell carcinoma of non-smokers and non-drinkers** | | | | | | | | |
| --- | --- | --- | --- | --- | --- | --- | --- | --- |
|  |  |  |  |  |  |  |  |  |
| *Clinical characteristics* | |  |  | *CD8/FoxP3 ratio ≥2.5* | | *CD8/FoxP3 ratio <2.5* | |  |
|  |  | *Total (n = 70)^+^* | | *(n = 33)* | | *(n = 37)* | | *p-value* |
| Age in years | Median (interquartile range) | 78.6 | (15.2) | 79.7 | (13.4) | 77.6 | (15.5) | 0.64 |
|  |  | *n* | *(%)* | *n* | *(%)* | *n* | *(%)* |  |
| Sex | Female | 59 | (84) | 29 | (88) | 30 | (81) | 0.52 |
|  | Male | 11 | (16) | 4 | (12) | 7 | (19) |  |
| T-stage | 1 | 17 | (24) | 9 | (27) | 8 | (22) | 0.90 |
|  | 2 | 24 | (34) | 12 | (36) | 12 | (32) |  |
|  | 3 | 5 | (7.1) | 2 | (6.1) | 3 | (8.1) |  |
|  | 4 | 24 | (34) | 10 | (30) | 14 | (38) |  |
| N-stage | 0 | 47 | (67) | 24 | (73) | 23 | (62) | 0.53 |
|  | 1 | 14 | (20) | 5 | (15) | 9 | (24) |  |
|  | 2 | 8 | (11) | 3 | (9.1) | 5 | (14) |  |
|  | 3 | 1 | (1.4) | 1 | (3.0) | 0 | (0) |  |
| M-stage | 0 | 66 | (94) | 31 | (94) | 35 | (95) | 1.0 |
|  | 1 | 4 | (5.7) | 2 | (6.1) | 2 | (5.4) |  |
| Recurrence | Yes | 18 | (26) | 8 | (24) | 10 | (27) | 1.0 |
|  | No | 52 | (74) | 25 | (76) | 27 | (73) |  |
| Hospital | Utrecht | 47 | (67) | 20 | (61) | 27 | (73) | 0.32 |
|  | Maastricht | 23 | (33) | 13 | (39) | 10 | (27) |  |
| Treatment | None | 2 | (2.9) | 0 | (0) | 2 | (5.4) | 0.54 |
|  | Radiotherapy | 5 | (7.1) | 2 | (6.1) | 3 | (8.1) |  |
|  | Surgery | 44 | (63) | 20 | (61) | 24 | (65) |  |
|  | Surgery with postoperative radiotherapy | 19 | (27) | 11 | (33) | 8 | (22) |  |
| Treatment intent | Curative | 63 | (90) | 31 | (94) | 32 | (87) | 0.43 |
|  | Palliative | 7 | (10) | 2 | (6.1) | 5 | (14) |  |
| ^+^, Patients were excluded when the TMA showed <10% tumor cells | | | | | | | | |
